# Comparison and analysis on sheep meat quality and flavor under pasture-based fattening contrast to intensive pasture-based feeding system

**DOI:** 10.5713/ab.21.0396

**Published:** 2022-01-21

**Authors:** Zhichao Zhang, Xiaoqi Wang, Yan Jin, Kai Zhao, Ziyuan Duan

**Affiliations:** 1 Key Laboratory of Adaptation and Evolution of Plateau Biota, and Laboratory of Animal Functional Genomics, and Qinghai Key Laboratory of Animal Ecological Genomics, Northwest Institute of Plateau Biology, Chinese Academy of Sciences, Xining 810000, Qinghai, China; 2 Institute of Genetics and Developmental Biology, Chinese Academy of Sciences, Beijing 100101, China; 3 Graduate School, University of Chinese Academy of Sciences, Beijing 100049, China; 4 Division of Energy Research Resources, The Dalian Institute of Chemical Physics, Chinese Academy of Sciences, Dalian 116000, Liaoning, China

**Keywords:** Branched-chain Fatty Acids, Fattening System, Fatty Acid Profile, Flavor, Hulunbuir Sheep, Slaughter Traits

## Abstract

**Objective:**

The objective of this study was to investigate the effect of 4-month intensive feeding on the meat quality, fatty acid profile, flavor, and growth performance of grazing Hulunbuir sheep (HBS).

**Methods:**

The HBS were selected 4-months after birth in a pasture rearing system as the experimental animals (n = 44, female, average body weight 23.8±2.2 kg) then divided equally into pasture-based grazing fattening (PAS) and concentrate-included intensive fattening (CON) groups for another 4-month finishing. When finished fattening, all animals were slaughtered to collect *musculus longissimus dorsi* subcutaneous adipose tissue and to investigate the influences on meat quality, fatty acid profile, flavor and growth performance.

**Results:**

The results showed lambs in CON group got significantly higher live weight, hot carcass weight, and dressing percentage. The CON group had significantly higher value of redness (a*), lightness (L*) and water holding capacity (p<0.05), significantly lower value of Warner-Bratzler shear force than the PAS group (p<0.05). The subcutaneous fat from CON group lambs demonstrated a significantly higher content of C18:1 and C18:2 (p<0.05), but lower C14:0 and C16:0, indicating an increased degree of unsaturated fatty acid. The content of 4-methyloctanoic acid, 4-ethyloctanoic acid and 4-methylnonanoic acid had increased 2 to 4 times, representing a more intense odor in the CON group. However, the values were still lower than most sheep breeds reported, indicating the indoor feeding system could not fundamentally deteriorate the excellent meat characteristic of HBS.

**Conclusion:**

It was evident that lambs in CON group exhibited a better meat production performance, improved in meat color, texture and healthier fatty acid profile through pasture-weaned concentrate included intensive fattening system, which offers a good alternative regimen for lamb finishing and has a wide prospection in the HBS meat industry.

## INTRODUCTION

Sheep are one of the important high quality human protein resources, sheep meat production has been steadily increasing globally along with the world purchase power parity in GDP per capita during the past 20 years, according to the Food and Agriculture Organization of United Nation (FAO) and the World Bank (WB) data set. To some extent, higher quantity of mutton consumption reflects a higher standard of living. This trend is pronounced in China as the consumption level of urban residents has continued growing based on the National Bureau of Statistics of China. The production of mutton in China has increased rapidly while the pork decreased gradually, beef increased slightly, especially after 2014. Moreover, consumers preferred the native lamb or mutton produced on pasture due to the perception that meat could be different and healthier when reared in extensive grazing on a variety of grasses, herbs and browse compared to products obtained from an indoor system [1]. However, what needs to be compromised is that overgrazing has been a severe problem worldwide, especially in the arid and semi-arid regions. To ensure the sustainable development of mutton sheep industry, China began to import forage products in large quantities after 2010, and the import scale has reached 550 million US dollars in 2019 (FAO). In addition, fattening lambs in feedlots with a concentrate-included diet after nursing their mother on pasture, i.e. intensive pasture-based fattening system is an alternative choice when there is limited grassland carry capacity and insufficient forage resources, in which lambs could be intensively fed after weaning with the advantage of accelerating commercialization and producing well-finished carcasses [2].

Hulunbuir, as part of the Eurasian steppe, with a total area of about 997.3×106 hm^2^, is an important animal husbandry region in China, where the Chinese indigenous sheep breed, Hulunbuir sheep (HBS), is raised as the main farm animal by both family farms and private-purpose production. Apart from be able to adapt the local harsh natural environment, the most outstanding characteristic of the HBS is incomparable meat quality with natural aroma with no unpleasant flavor. The problem faced currently in Hulunbuir mutton sheep industry, is the double restrictions of overgrazing and insufficient forage, which calls for profound changes in practices to adopt the indoor system with the pasture grazing benefit [3]. Although fattening lambs with concentrate supplement is known to increase carcass yield, fatness and growth performance, fattening lambs on pasture was recognized as significantly affecting the sensory properties of the cooked meat [4], and leading to greater deposition of conjugated linoleic acid (CLA) which is an essential nutrient for health compared to grain-finishing lambs [5,6].

Recent studies showed that besides growth rate and car cass characters the production system might also affect the meat quality [7]. On the issue, there is inadequate information on the effect feeding regimens have on sheep meat quality especially in flavour, and little is known about the effect of combining of high quality pasture with concentrates on HBS. Whether the concentrate-based finishing would injure the meat flavor of HBS is acknowledged as an important issue, which would influence the acceptability of the sheep meat products [8,9].

The aim of this study was to comprehensibly compare sheep meat quality between the pasture finishing and concentrate-included fattening after pasture weaned for HBS. Specifically, focus on changes in slaughter traits, physical measurements of meat quality, fatty acid profile and the content of compounds responsible for flavor. The results are imperative to formulating improved sheep meat production in Hulunbuir.

## MATERIALS AND METHODS

### Animals and finishing system

The experiment conducted conformed to the guidelines by the National Institute of Animal Health, China (GB 14925-2001), and outline of Bayan Farm in Inner Mongolia Autonomous Region (License number BY-18072-01). The ewes of HBS are seasonal estrus and all their lambs were born as single in mid-March each year. In the study, a total of 44 weaned, around 4-month old HBS female lambs were randomly selected from a herd of about 1,000 heads in HulunBuir grassland. The lambs selected with an average body weight (BW) of 23.8±2.2 kg were labelled and separated into two groups at equal numbers of 22 in each and offered the pasture-based grazing fattening system (PAS) and concentrate-included intensive fattening system (CON). The experiment was conducted at State-operated Bayan Farm in Inner Mongolia Autonomous Region, China, and lasted for 4 months until slaughtering.

#### Pasture-based grazing fattening system

Lambs in the group were separated from their mothers gradually in one week when arriving at weaning age (4-month old) and grazed on pasture in different flocks during daytime and kept in the sheepfold in the night as a traditional feeding program. Water was accessed at the designated time of noon and dusk daily.

#### Concentrate-included intensive system

Lambs in the CON group were transported to Bayan farm and allocated to sheep pen (10 m×3.5 m) after weaned at 4-month old with pasture grazing. The total mixed ration (TMR) was offered, consisted of reaped hay, silage corn and concentrate with the proportion of 20%:47%:33%, which was as same as the local commercial meat sheep. Fresh clean water was accessed freely during the finishing period.

The chemical composition and fatty acid profile of forage grass and the TMR used in this study were analyzed by Feed Potency and Safety Supervision, Inspection and Testing Center (Beijing, China) of The Ministry of Agriculture and Rural Affairs after 24 hours freeze-drying pretreatment, and the results are shown in Table 1.

### Slaughter procedures and samples collection

All lambs were slaughtered at the age of 8-month as local finishing habit following the principles and outlines concerning feeding and slaughtering of Bayan Farm. Live weight (LW) of each lamb was measured after 24 h fasting on the previous day at the end of the experiment, hot carcass obtained after slaughtered when separation of feet, skin, head, lungs and trachea, heart, liver, spleen, and gastrointestinal tract. The dressing percentage (DP) was calculated as the proportion of hot carcass weight (HCW) on LW. Then, the carcasses were cut lengthwise in two equal parts and *musculus longissimus dorsi* (LD) was removed integrally from the right half, then transported to the laboratory at 4°C within 24 hours for the meat quality traits determination. The subcutaneous adipose tissue between the ribs of the 12th and 13th were collected with 50 g, packed with plastic bags into cryogenic storage box and transported to the laboratory with dry-ice, where the samples were stored at −20°C until the analyses for fatty acid profile and flavor related compounds.

### Measurements of meat quality

#### Physical measurements of determination

The pH value of the meat samples was measured initially as pH_1_ and 24 hours later, denoted as pH_24_, with a handheld piercing pH meter (Testo 205; Shenzhen Detu Co., Ltd., Shenzhen, Guangdong, China) after acid discharge in 4°C in the same position. The color of the *longissimus lumborum muscle* was determined in 24 hours after slaughter by colorimeter (Konica CR400; Konica Minolta Sensing, INC., Tokyo, Japan) using the CIE L*, a*, b* system, in which L* (lightness) describes the relationship between reflected and absorbed light without a specific wavelength. Positive a* means red and negative one shows green. Positive b* means yellow and negative one is blue.

To determine the cooked meat percentage (cooking loss), about 100 g muscle sample (m1) was placed in polyethylene bag in a water bath at 72°C, when the internal temperature of the sample reached 70°C the sample was removed to room temperature for cooling, blotted dry and reweighed (m_2_), then cut into 2×2×1 cm pieces. Cooked meat percentage was calculated as m2 ⁄m1×100% (Honikel [10]). Tenderness was evaluated in triplicate by Warner-Bratzler shear force (WBSF) as described before [11]. The meat pieces were sheared with the muscle fiber direction perpendicularly using tenderness analyser (C-LM3B; College of Engineering, Northern east Agricultural University, Harbin, China) equipped with a WBSF device. The texture analyzer was set with a crosshead distance of 25 mm with a speed of 60 mm/min.

Water holding capacity (WHC) was assessed by determin ing the mill loss, as the sample was sliced to 1 cm thickness, using a circular sampler with a diameter of 2.523 cm to collect the sample and weigh it as m_3_. The circular sample was placed between two layers of filter paper followed by 18 layers of filter paper on the top and bottom each, put on the pl atform of press meat instrument (RH-1000; Run Hu Instrument INC., Guangzhou, China) with a pressure of 35 kg for 5 minutes, then weight the sample immediately as m_4_. The WHC was obtained as (m_3_−m_4_)/m_3_×100%.

Moisture content (MC) and intramuscular fat (IMF) were determined using the Soxhlet procedures as described before (Holman [12]). Well-defined 5 to 8 g minced meat samples (m_5_) were full mixed and put into an aluminium vessel, mixed with sea sand and ethanol, dried for 4 hours at 105°C in drying oven, followed by cooling to room temperature in a desiccator and weighed (m_6_). The MC was calculated as (m_5_−m_6_)/m_5_×100%. The dried samples were then transferred into the Soxhlet equipment, the extraction was carried out with petroleum ether for 6 hours at 60°C. After the evaporation of the solvent, the flask (weight as m_7_) with the fat was dried for 1 hour at 100°C, then cooled to room temperature in a desiccator and weighed (m_8_). Intramuscular fat content was calculated as Fat (%) = (m_8_−m_7_)/m_5_×100%.

#### Fatty acid profile

Procedures of solid-phase microextraction (SPME) were used as described previously [13] and fatty acids were identified using a gas chromatography-mass spectrometry (GC-MS). Briefly, a portion of (0.05 to 0.1 g) fat samples were removed from the −80°C and transferred into a 20 mL headspace vial, added 2 mL n-hexane and 4 mL acetylcholine-methanol (1:10, v/v). The sealed vial was heated in a water bath at 80°C for 2 hours and shaken every 20 minutes. When cooled to room temperature, add 5 mL 7% Na_2_CO_3_ (m/v) and shaken to homogenous. Undissolved substances were removed after cooling. The solution was centrifuged at 3,000 g in 5 min, the organic phase absorbed, then the water phase was extracted with 2 mL n-hexane once more. The two organic phases were combined and n-hexane used to bring to a constant volume of 5 mL. Then, the solution was filtered with a 0.22 μm organic phase filtration membrane, stored at −20°C for analysis.

The solution prepared was performed using a 7890A GC with 7000B Triple Quadrupole MS GC/MS (Agilent Technologies Inc., Palo Alto, CA, USA), fatty acids were separated on a fused silica DB-5MS capillary column (30 m×0.25 mm ×0.25 μm; Agilent Technologies Inc., USA), helium was used as a carrier gas with a flow rate of 1.5 mL/min. The injection mode used as, the split was set to 30:1 ratio, solvent delay for 9 min. The initial GC oven temperature was set at 60°C, held for 2 min, followed increased to 260°C at a rate of 5°C/min and finally 20°C/min to 300°C, held for 5 min. The high sensitivity of electron ionization (EI) ion source was at 230°C with the quadrupole temperature set at 150°C. The mass spectrometer (MS) operating conditions were set as positive EI mode (EI +) using automatic gain control with 70 eV of electron energy. MS operated in full scan and selected-ion monitoring mode to record the abundance of the ions which was listed in Supplementary Table S1. The data was collected with Agilent mass hunter workstation software.

Volatile compounds were identified either by comparing their mass spectra and retention time with those from standard compounds (Sigma-Aldrich Corp., St. Louis, MO, USA) or by comparing them with those contained in a mass spectra library (Thermo Electron Corp., Waltham, MA, USA) with a similarity index of 800 or greater.

Flavor related compounds assay: When the flavor related compounds of 4-methylphenol (MP) and 3-methylindole (MI) were determined, the procedure above was adjusted slightly. A portion (0.05 to 0.1 g) of the fat samples were transferred into a 20 mL headspace vial, sealed, and inserted into an SPME extraction needle. After heating at 60°C to 90°C for 15 to 50 min in a water bath, the samples were directly injected with the extraction needle and analyzed by GC-MS. The chromatographic column for separation was DB-WAX (30 m×0.25 mm×0.25 μm; Agilent Technologies Inc., USA), helium was used as a carrier gas with a constant flow rate of 1.5 mL/min without solvent delay. The oven temperature was initially held at 50°C for 1 min, and increased to 110°C at a rate of 10°C/min, followed by 5°C/min to 230°C, then held for 5 min. The MS operating conditions and data collection were set as same as aforementioned.

### Statistical analysis

Statistical analyses were performed with Excel Pro 2016 (Microsoft Inc., Seattle, WA, USA), significant effects were determined at p<0.05. The principal component analysis (PCA) was carried out through SPSS 20.0 (IBM, Armonk, NY, USA), and Past V3.10 for data standardization and calculation respectively, which was to evaluate the eating quality attributes and illustrate the relationship among variables and samples.

## RESULTS

### Slaughter traits and physical meat quality characters

The effects of the finishing on lamb performance and slaughtering traits are presented in Table 2. The indoor fattening system had a significantly increase (p<0.05) over the pasture finishing system either in LW or in HCW within the same periods. Hence, the lambs in the CON group had a higher DP (p<0.001) and an attenuated range of variation in BW. Comparing the physical meat quality measurements between PAS and CON groups, the lightness (L*), redness (a*), and WHC were significantly (p<0.05) increased except mill lose (ML), WBSF were significantly decreased (p<0.05) while the values of pH_1_ and pH_24_ were significantly declined (p<0.001 and p<0.05) in CON group, but all within the normal range of values. The other parameters like yellowness (b*), dropping loss, MC and cooked meat percentage were similar in values between the two groups. Although the IMF was slightly enhanced in CON lambs but did not reach the statistically significant level (p>0.05). In overall view to the physical parameters, the meat was more red, bright, and tender, and the meat quality was greatly improved by the indoor finishing.

### Fatty acid profile for subcutaneous fat

By the method in the study, 8 types of main fatty acids in the subcutaneous adipose tissue of the sheep were identified. They were myristic acid (MA, C14:0), palmitic acid (PA, C16:0), stearic acid (SA, C18:0), palmitoleic acid (C16:1), oleic acid (OA, C18:1), and linoleic acid (LA, C18:2) as given in Table 3. Compared to the PAS group, the CON group had almost the same content of saturated fatty acid (SFA) (p>0.05), but a higher value of unsaturated fatty acid (USFA, p<0.05), which was embodied by the significantly lower content of MA (p< 0.01) and highly significantly lower in PA (p<0.001). In contrast, the CON group had a significantly higher content of OA and LA (p<0.001), therefore, the increased ratio of USFA/SFA indicates that unsaturated level was enhanced in subcutaneous fat in the CON group.

### Flavor related compounds in subcutaneous fat

From Table 4, it was clear the fattening system had a significant influence on the content of flavor related compounds in the sensory attributes of different production systems. Except that the content of MP was highly declined in the CON group (p<0.01), the branched-fatty acids (BCFAs) like 4-methyloctanoic acid (MOA), 4-ethyloctanoic acid (EOA) and 4-methylnonanoic acid (MNA) were significant augmented (p<0.001, p<0.01, p<0.05 respectively). Although MI also increased with a large amount in the CON group, it did not reach the significant level (p>0.05).

### The principal component analysis for the eating quality

**Figure 1:**
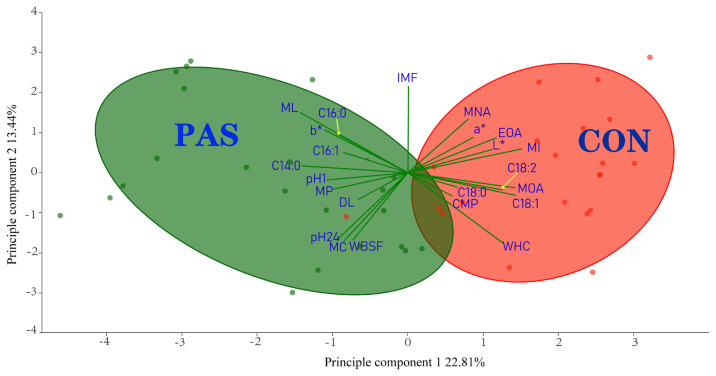
The top 2 principal components of the principal component analysis (PCA) for the sheep meat eating quality of Hulunbuir sheep under different production systems. The green lines represent the parameter vectors, the larger of the modulus of the vector means the greater of the interpretation degree of this parameter to the variation of sheep meat eating quality. The projection length of the vector on the x-axis means its correlation with principal component 1, and the projection length of the vector on the Y-axis means its correlation with principal component 2, the longer of the projection length means the stronger of the correlation. The angle between two vectors represents the correlation between two indicators. If the angle is acute, the smaller the angle is, the stronger the positive correlation is. While the angle is in obtuse, the negative correlation would be stronger as the larger of the angle. PAS, pasture-based system; CON, concentrate-included system; L*, lightness; a*, redness; b*, yellowness; ML, mill lose; WHC, water holding capacity; DL, dropping loss; MC, moisture content; WBSF, Warner-Bratzler shear force; CMP, cooked meat percentage; IMF, intramuscular fat; pH_1_, the pH value within 1hour postmortem; pH_24_, the pH value within 24 hours postmortem; MOA, 4-methyloctanoic acid; EOA, 4-ethyloctanoic acid; MNA, 4-methylnonanoic acid; MP, 4-methylphenol; MI, 3-metlylindole.

Figure 1 presents the result of PCA from the data among physical meat quality characters, fatty acid profile and flavor related compounds. The first two principal components accounted for 36.25% of the total variance (Supplementary Table S2) with 22.81% and 13.44% contributions by PC1 and PC2 respectively. Among these detected indicators, MI, MOA, OA, and WHC had a greater positive effect on PC1, while MA and ML had a much negative effect on the PC1. For PC2, IMF and yellowness (b*) had a greater positive effect, while pH_24_, WHC, MC, and WBSF acted in greater negative role.

## DISCUSSION

Although the sheep meat production systems varied from diversified pasture to feedlot by grain feeding among production countries, feeding with grain based rations in confined barn has been a trend for finishing lambs, especially for temperate-cultivated pastures, in which intensive grazing or grazing plus supplementation were usually adopted. This system is already very common in dairy cow and beef cattle, but relatively less in mutton production because of variety of conditions in sheep, like different digestive physiology and difference in forage quality preferences [14]. The diets of feeding strategies designed with verification were not recommended for sheep nutrition and were not abundant as those for dairy and beef cattle [15]. It was necessary to formulate suitable sheep meat production system conditioned to local climate and grass management.

In this fattening study, lambs in the CON group presented higher LW and HCW than ones grazing on pasture in the same periods, followed by a higher DP, indicating that concentrate supplementary fattening in a sheep pen after pasture weaned exhibit a better meat production performance. The result was consistent with the other similar researches about indoor fattening experiments [16] and also in accord with the result of pasture grazing steers, which may be less effective than other diets for beef production because of a decrease in daily gain and low productivity [17]. The difference in growth performance between the two finishing systems suggested that changes in the rearing environment and forage affected the growth performance profoundly. Compared to the condition of the indoor feeding system, the outdoor sheep will spend more time walking due to greater available space and to investigate the new environment, thus consuming additional energy.

Generally, the physical measurements for meat quality ef fected by different production systems related to the levels of gross energy (GE), crude protein (CP) in forage and interaction with the genetic propensity of animals for muscle and fat deposition [4]. The lambs fed in indoor system would produce meat with higher fat content than those with forage based diets at the same LW [18]. In the present study, we observed higher fat content in the carcass of the CON group during the slaughter process because of the higher GE and CP of TMR than the mixed grass in the pasture (Table 1).

According to the perception of consumers, meat color is an important and visual mark used to estimate the quality and freshness of sheep meat at the point of purchase [19]. Unlike lightness and yellowness, redness has a stronger correlation with meat acceptance. When the value of redness is above 14.5, the fresh meat is accepted by above 95% of consumers [20]. In the current study, the mean value both in PAS and CON reached 19.85 and 22.56 respectively, demonstrating the excellent marketing appearance of Hulunbuir lamb. In terms of lightness, the lambs in the CON group presented very significant higher value than that in PAS. It was also in line with the previous studies which had reported higher meat lightness in lambs raised on concentrate-based system than the counterpart in the pasture [21]. Being the color of meat was affected by fat deposition and oxidation process [5], the reasons for a higher lightness of lambs in CON can be attributed to the more fat content within IMF even though the difference did not reach a significant level, and might be induced by less oxidation in metabolism process due to more idling time and less physical activity in housing than outdoors [22]. This inference was also in keeping with the observation of Caneque et al [23], who had noted a higher lightness value in LD of dry lot lambs compared to pasture lambs and also considered it to be due to a different physical activity level.

The ultimate meat pH values (pH _24_) of the two groups were between 5.7 to 5.9 which could be considered as the acceptable quality range [24], but the lambs from PAS group had a significantly higher pH value than ones from CON group (p<0.05). Some studies have showed that the ultimate meat pH of lambs fed in extensive conditions is higher than that of lambs subjected to intensive fattening [25]. By contrast, other studies have reported that different feeding systems had a limited or no effect on meat pH value in sheep and goats [15,16]. Apparently, our study was in line with the former observations. Before those inconsistent observations above, a study found that cattle fed low energy rations had lower muscle glycogen contents when compared to one fed high energy rations [26]. A subsequent study reported that high energy rations had a positive effect on glycolysis and post mortem reductions in pH by increasing muscle glycogen reserves and fat thicknesses in sheep and cattle carcasses [27]. Hence, the difference in the value of pH_24_ to differences in muscle glycogen reserves and energy levels was connected to feeding systems, low muscle glycogen reserve associated with low energy supply [28]. Compared to the energy intake of the CON group, the lambs in the PAS group gained less energy because of lower GE in the ration (Table 1), and muscle glycogen reserves would be depleted more quickly. We speculated that the lower deposition of glycogen reserves in muscle from PAS groups was the reason for high pH value. In addition, WHC may be affected by rations. Our study was also in accord with the previous reports that lambs fattened intensively with concentrate to have higher WHC than ones finished on pasture [29].

The tenderness of meat is another primary consumption character and is affected by the IMF directly. Even though there was no significant difference in IMF between the lambs from CON and PAS group, a higher value was observed in CON lambs, and the meat fed with forage inclusion with concentrate was more tender indicated by the significantly lower value of WBSF. This observation was also consistent with the previous reports [16]. Many studies have reported that the fatness of carcass influences the WBSF and it decreases with the increase of carcass fatness because of the reduction of connective tissue strength [30,31]. Meanwhile, some studies reported that a greater growth rate of lambs may produce an increase in soluble collagen and then result in more tender meat [32]. In the present study, lambs in CON had a higher growth rate than that in PAS and had a higher content of IMF, which may be a feasible explanation for more tender in CON lambs.

It is a consensus that consumers of sheep meat usually place the most weight on flavor followed by tenderness and juiciness [4], therefore, fatty acid profile plays an important role in the definition of meat quality as well as with differences in organoleptic attributes, like taste and undesired sensory characteristics [33]. Many reports showed that the feeding system could alter the fatty acid profile on beef and lamb [5–7]. Our study has substantiated it again from subcutaneous fat other than from muscle. From the results presented in the study, the most abundant fatty acid was OA (C18:1) (31.63% to 36.91%), then PA (C16:0, 24.61% to 27.54%) and SA (C18:0, 18.84% to 20.65%) in both groups of production systems. The total percentages of the three dominant fatty acids in the CON and PAS group were 78.01% and 82.17% respectively, which were in line with the data of previous research [34–36].

For ruminants, the forage degradation in their rumen is a complex and special process involving multiple activities with microorganisms, where the microbiota synthesize long chain n-3 fatty acids through their enzymatic action from the α-linolenic acid precursor when in a pasture feed system is offered. However, the biohydrogenation pathway affected through shifting *trans*-vaccenic acid production to C18:1 *trans*-10 when foraging includes a concentrate or grain finished either in lamb or in beef [37–39]. As shown in Table 1, the pasture has a higher content of C18:3, and this might be the reason that both OA (C18:1) and LA (C18:2) were highly significantly increased in the CON group compared to PAS group in our study. We then speculated there was intensive hydrogenation activity through microbiota in the rumen which was responsible for the formation of higher USFA like CLA while when forage includes a concentrate the result is a significantly higher value of USFA than pasture finished system, and in turn, improving the sheepmeat quality to benefit the human health. It is a plausible explanation that SFA is inclined to decrease in the CON group because the total amount of fatty acid is limited and would favor producing more USFA, resulting in a significantly decreased amount of SFAs like MA (C14:0) and PA (C16:0). Apparently, lambs finished with forage inclusion concentrate had a better fatty acid profile compared to the lambs merely pasture feeding.

The formation of short chain BCFAs was regarded as the main contributor to mutton flavour, which is the species-specific odor and the most important factor for consumers accepting lamb or mutton [4]. A recent study reported that BCFAs were positively correlated with the indexes such as consumer lamb flavor intensity scores, flavor liking, overall flavor impact and odor impact, which means the higher amount of BCFAs the lower acceptability of consumers [9]. The most notable BCFAs were MOA, EOA, and MNA, present in ovine fat and associated with “mutton” flavour [4,13]. Other compounds, such as MP and MI, accumulate in fat reservoirs and were associated with distinct “pastoral” flavor in sheepmeat [40]. Both “mutton” and “pastoral” flavor were found to be undesirable odor recognized by consumers, and higher concentrations of these compounds have been observed when receiving a grain based finishing diet [40]. In the current study, with concentrate inclusion in the ration, the content of MOA in lambs was above 4-fold higher than that of PAS lambs, and EOA was above 2-fold. While MNA could not even be detected in lambs of the PAS group but was observed in lambs of the CON group by a mean value of 20.87 μg/g. Apparently, whether MOA, EOA, or MNA have increased several times in the group of CON, which was in agreement with the observation aforementioned [4]. Compared with the lambs in PAS, lambs in CON resulted in intensified “mutton” flavor.

As BCFAs, MOA, EOA, and MNA can be *de novo* synthesized in adipose tissues [41]. In general, fatty acid synthesis is primarily mediated by the acetyl CoA carboxylase (ACC) and fatty acid synthase (FASN), whereby acetyl-CoA is utilized by FASN to initiate a new acyl chain, is subsequently elongated using malonyl-CoA generated by ACC [42]. Moreover, FASN is able to incorporate methylmalonyl-CoA or ethylmalonyl-CoA units instead of malonyl-CoA to produce methyl or ethyl branches on even-numbered carbon atom in the less efficient condition [43]. Methylmalonyl-CoA and ethylmalonyl-CoA are then produced in the cytosol as side-products by ACC using propionyl-CoA and even butyryl-CoA [44]. For ruminants, the rumen microbes can produce volatile fatty acid (VFA) including propionate and butyrate, which are chiefly absorbed by the epithelium of the rumen and of the omasum [45]. From the data of Table 1, the diet of CON group contained more than 3-fold ether extract (crude fat) than that of PAS, which might be a primary factor for the higher content of BCFAs in the CON group.

Since the amount of MP and MI in the subcutaneous fat of HBS was too low to be detected accurately, their results, therefore, were demonstrated with the relative amount. Although there was some difference between MP and MI in CON and PAS lambs, it didn’t make a lot of sense, as the lambs from two different finish systems had very low levels of MP and MI, which means a very low level of “pastoral” flavor.

Looking through related studies, Frank et al [9] reported that their experimental lambs with HCW from 16.6 to 20.2 kg, being close to the HCW of the present research, had the content of MOA, EOA, MNA, MP, and MI were 220 to 393, 89 to 170, 35.1 to 63.6, 36.0 to 89.2, 32.9 to 118.9 μg/g, respectively [9]. The MOA, EOA and MNA from the lambs of Merino sheep were reported 86, 24, 18 μg/g respectively, and the content of these three compounds in the crossbreed of Merino and Ile de France were 49, 26, and 5.6 μg/g, respectively [46]. Schiller et al. had reported the content and distribution of these 3 compounds among 6 breeds, ranging from 56.9 to103, 13.3 to 19.7, and 15.6 to 46.6 μg/g, respectively [47]. The data above are much higher than the counterparts from the lambs in the CON group, indicating that even if the content of compounds responsible for the unpleasant flavor in the CON group were higher than in the PAS group, they were still lower than most breeds reported. In other words, the odor of HBS would be intensified when concentrate inclusion in feedstuff fed in pens, but it could not damage the excellent characteristics of HBS in flavor fundamentally. Whether these differences in the content of BCFAs could be used as an indicator to distinguish the meat of HBS breed, still needs extensive investigation.

As known, the eating quality of sheep meat covers a wide range of complex traits. In the present study, there were 23 traits involved in detection. Each trait in two production systems has a unique embodiment, how to simplify comprehensive evaluation and select reduced traits as a marked character was in great request. Principal component analysis is therefore an optional consideration in which multivariate statistical analysis can reduce the complexity of the issue. Through this method, the lambs in the study were separated into two parts primarily by the finishing systems they received (Figure 1), it was then possible to understand the commonality and difference clearly regarding the eating quality from different production systems, and Figure 1 supplied the information that sheep meat from the CON group is ruddier, lighter, with a higher content of compounds related with unpleasant flavor and USFAs compared to the meat from PAS group.

## CONCLUSION

In the present study, lambs from two feeding regimens of pasture grazing and concentrate-included feeding in sheep pen were conducted for systematic comparison, especially in flavor character with GC-MS instead of trained sensory panels. The results embodied changes in the rearing environment and forage, which affected the growth performance, meat physical measurements and altered the fatty acid profile profoundly. Taking the fattening system of TMR after weaned in pasture exhibited a higher growth rate in meat productivity, improved eating quality symbolized by redder and brighter in color, tender in taste and better fatty acid profile for human health. Despite the concentrate inclusion feeding system increasing the content of major BCFAs significantly in HBS, it was still lower than most sheep breeds and did not fundamentally deteriorate the excellent characteristic of HBS.

In conclusion, it was evident that the pasture-weaned in tensive fattening system (CON group) offers a good alternative regimen for lamb finishing production, which takes the advantage of both pasture grazing and confined intensive feeding systems and has a wide prospection for extension and application in the HBS meat industry.

## Figures and Tables

**Table 1:** The chemical composition and fatty acid profile of total mixed ration and pasture offered in the study (Freeze-dried samples)

Items	TMR^1)^	Pasture^2)^
Moisture (%)	41.80	16.60
Crude protein (%)	13.86	11.12
Ether extract (%)	4.90	1.30
Ash (%)	13.25	5.60
NDF (%)	39.23	52.07
ADF (%)	20.85	27.94
GE (MJ/kg)	17.76	14.32
C12:0 (mg/g)	0.1	-
C13:0 (mg/g)	0.1	-
C14:0 (mg/g)	0.1	0.2
C16:0 (mg/g)	8.5	2.3
C16:1 (mg/g)	0.1	-
C17:0 (mg/g)	0.1	-
C18:0 (mg/g)	1.1	0.3
C18:1n9c (mg/g)	13.9	0.8
C18:2n6c (mg/g)	24.7	2.7
C18:3n3 (mg/g)	1.9	5.3
C20:0 (mg/g)	0.3	0.2
C20:1 (mg/g)	0.2	0.1
C22:0 (mg/g)	0.3	0.1
C22:1n9 (mg/g)	0.1	-
C23:0 (mg/g)	0.1	-
C24:0 (mg/g)	0.4	0.2

NDF, neutral detergent fibre; ADF, acid detergent fibre; GE, gross energy.

1) TMR, total mixed ration, the ratio of hay, silage and concentrate is 20%:47%:33%.

2) The vegetation types of Hulunbuir grassland are *Stipa baicalensis* and *Leymus chinensis*, the main accompanying grass are *Artemisia frigida*, *Poa annua*, *Medicago falcata L*..

**Table 2:** The slaughter traits and meat quality measurements with different production systems in Hulunbuir sheep (mean±standard deviation)

Characteristics	PAS	CON	Significance level
LW (kg)	34.39±5.08	38.11±5.50	^*^
HCW (kg)	15.70±3.33	19.45±2.80	^***^
DP (%)	45.36 ± 3.71	51.06± 1.59	^***^
L*	36.07±2.35	39.24±0.74	^***^
a*	19.85±1.79	22.56±1.55	^***^
b*	6.81±1.40	6.33±0.45	NS
ML (%)	31.1±4.19	28.6±3.18	^*^
WHC (%)	58.51±5.61	61.57±4.31	^*^
DL (%)	1.29±0.57	1.33±0.33	NS
MC (%)	74.98±1.16	74.46±1.17	NS
WBSF (N)	71.28±25.84	55.55±20.84	^*^
CMP (%)	85.25±4.01	84.58±3.4	NS
IMF (%)	3.45±0.77	3.61±0.83	NS
pH_1_	6.61±0.16	6.36±0.18	^***^
pH_24_	5.87±0.08	5.78±0.18	^*^

PAS, pasture-based system; CON, concentrate-included system; LW, live weight; HCW, hot carcass weight; DP, dressing percentage; L*, lightness; a*, redness; b*, yellowness; ML, mill lose; WHC, water holding capacity; DL, dropping loss; MC, moisture content; WBSF, Warner-Bratzler shear force; CMP, cooked meat percentage; IMF, intramuscular fat; pH_1_, the pH value within 1 hour postmortem; pH_24_, the pH value within 24 hours postmortem.

NS, no significant;

* p<0.05;

** p<0.01;

*** p<0.001.

**Table 3:** The fatty acid profile of different production systems in Hulunbuir sheep (mean±standard deviation)

Characteristics	PAS	CON	Significance level
MA (C14:0, %)	7.05±2.45	4.81±1.18	^**^
PA (C16:0, %)	27.54±2.39	24.61±1.82	^***^
SA (C18:0, %)	18.84±2.73	20.65±3.14	NS
POA (C16:1, %)	1.97±0.85	1.62±0.39	NS
OA (C18:1, %)	31.63±2.69	36.91±3.33	^***^
LA (C18:2, %)	2.00±0.49	3.52±0.54	^***^
SFA (%)	58.03±3.08	57.06±3.55	NS
USFA (%)	39.81±2.81	42.47±3.51	^*^
USFA/SFA	0.69	0.74	

PAS, pasture-based system; CON, concentrate-included system; MA, myristic acid; PA, palmitic acid; SA, stearic acid; POA, palmitoleic acid; OA, oleic acid; LA, linoleic acid; SFA, saturated fatty acid; USFA, unsaturated fatty acid.

NS, no significant;

* p<0.05;

** p<0.01;

*** p<0.001.

**Table 4:** The flavor related compounds of the different production system in Hulunbuir sheep (mean±standard deviation)

Characteristics	PAS	CON	Significance level
MOA^1)^ (μg/g)	4.48±6.99	19.68±14.93	^***^
EOA^1)^(μg/g)	8.22±7.06	17.46±12.12	^**^
MNA^1)^ (μg/g)	0	20.87±22.58	^*^
MP^2)^	553.87±533.43	134.56±111.43	^**^
MI^2)^	185.33±76.51	313.47±154.01	NS

PAS, pasture-based system; CON, concentrate-included system; MOA, 4-methyloctanoic acid; EOA, 4-ethyloctanoic acid; MNA, 4-methylnonanoic acid; MP, 4-methylphenol; MI, 3-metlylindole.

1) The content was an absolute quantification result.

2) The content was a relative one (peak height/sample mass).

NS, no significant;

* p<0.05;

** p<0.01;

*** p<0.001.

## SUPPLEMENTARY MATERIAL

**Supplementary Table S1**. Time windows for GC-MS measurements **Supplementary Table S2**. The Eigenvalue of the top 10 principal components and the proportion of variance
