# Supplementary material for: Comparison and analysis on sheep meat quality and flavor under pasture-based fattening contrast to intensive pasture-based feeding system
Source: Anim Biosci. 2022 Jan 21;35(7):1069–79. doi: 10.5713/ab.21.0396 (PMC9271384; doi:10.5713/ab.21.0396)
Supplement: Supplementary file 1 [file ab-21-0396-suppl.pdf]

Supplementary

Table S1. Time windows for GC-MS measurements

Time window	Start Time. min	Mode	Mass range, m/z
1	0	Solvent delay	null
2	9	Full scan mode	50-400
3	19.5	Selected ion mode	74,87,99,115,123,141,172
4	21.5	Selected ion mode	55,74,87,101,114,115,125,129,143,155,169,186
5	27	Full scan mode	50-400

Table S2. The Eigenvalue of the top 10 principal components and the proportion of variance

Principal components (PC)	Eigenvalue	The proportion of variance, %	Accumulated proportion, %
1	4.84786	22.807	22.807
2	2.85753	13.443	36.250
3	2.67043	12.563	48.813
4	1.87911	8.8404	57.6534
5	1.6588	7.804	65.4574
6	1.36697	6.4311	71.8885
7	1.06932	5.0307	76.9192
8	0.87182	4.1016	81.0208
9	0.794402	3.7373	84.7581
10	0.709124	3.3361	88.0942
